# A role for aberrantly expressed nuclear localized decorin in migration and invasion of dysplastic and malignant oral epithelial cells

**DOI:** 10.1186/1758-3284-3-44

**Published:** 2011-09-29

**Authors:** Nyla Dil, Abhijit G Banerjee

**Affiliations:** 1 Departments of Oral Biology, University of Manitoba, Health Sciences Center, Winnipeg, Canada; 2 Immunology, University of Manitoba, Health Sciences Center, Winnipeg, Canada; 3 Medical Microbiology and Infectious Diseases, University of Manitoba, Health Sciences Center, Winnipeg, Canada; 4 Center for Genomic Bio-Medicine and Res. Inst, Durg, Chhattisgarh, India

**Keywords:** Oral Cancer, decorin, functional genomics, shRNA, invasion, migration, IL-8, EGFR

## Abstract

**Background:**

Oral cancer is the sixth most common malignancy worldwide with a mortality rate that is higher than many other cancers. Death usually occurs as a result of local invasion and regional lymph node metastases. Decorin is a multifunctional proteoglycan of the extracellular matrix that affects the biology of various types of cancer. Previously; we have shown that decorin is aberrantly expressed in the nucleus in human dysplastic oral keratinocytes (DOK) and malignant squamous cells carcinoma (SCC-25) and human biopsy tissues. In this study, we examined the role of nuclear decorin in oral cancer progression.

**Materials and methods:**

We have used a post-transcriptional gene silencing (RNA interference) approach to stably knockdown nuclear decorin gene expression in DOK and SCC-25 cells using a specific shRNA plasmid and a combination of immunological and molecular techniques to study nuclear decorin function in these oral epithelial cell lines.

**Results:**

More than 80% decorin silencing/knockdown was achieved as confirmed by real time PCR and western blot analysis in both DOK and SCC-25 cells. This RNA interference-mediated knockdown of nuclear decorin expression resulted in significantly reduced invasion and migration in these cell lines as measured by Matrigel™ coated and uncoated Trans well chamber assays respectively. Decorin silencing also resulted in reduced IL-8 mRNA and proteins levels in these cell lines. Culturing decorin silenced DOK and SCC-25 cells, with recombinant human IL-8 or IL-8 containing conditioned medium from respective un-transfected cells for 24 h prior to migration and invasion experiments, resulted in the salvation of reduced migration and invasion phenotype. Furthermore, we found that nuclear localized decorin interacts with EGFR in the nuclear fractions of both DOK and SCC-25 cells. Interestingly, EGFR (trans) activation has previously been shown to be involved in IL-8 production in various epithelia.

**Conclusions:**

Taken together, our results indicate that nuclear localized decorin plays an important role in migration and invasion of oral cancer cells and thus may present as a novel potential target for the treatment of oral cancer.

## Background

Oral squamous cell carcinoma (SCC) is the sixth most common cancer in the world [1,2]. It accounts for roughly quarter of a million newly diagnosed cancers worldwide, and is the most frequently diagnosed cancer in developing countries of the world [3,4]. Despite improvements in surgical techniques, radiation therapy protocols, and chemotherapeutic regimens [5], the overall five year survival rate for oral SCC remains at 50% and has not significantly improved in the past 30 years. Morbidity associated with the disease further affects the quality of life in patients as nutrition, speech and general immunity gets compromised as well. In oral cancer patients, death usually occurs as a result of local invasion into the stromal tissue of head & neck and cervical lymph node metastases [6,7].

Decorin is a member of small leucine-rich repeat proteoglycans (SLRPs) family and is primarily synthesized by fibroblasts and myofibroblasts [8]. Members of SLRPs family are structurally related and play major roles in the organization of the extracellular matrix (ECM) and the regulation of cell behaviour [9]. SLRPs have a 40-50 kDa protein core with a central leucine rich repeat (LRR) domains characterized by a common molecular architecture adapted for protein-protein interaction [10]. Decorin is a known ligand for epidermal growth factor receptor (EGFR) [11,12].

Decorin is normally present in the extracellular stromal compartment and has been shown to restrain growth of many tumor cells [13] by down-regulating the epidermal growth factor receptor (EGFR) [9]. The underlying mechanism is not fully understood but includes direct decorin binding to EGFR followed by sustained internalization of EGFR via caveolar-mediated endocytosis [14] and the apoptosis triggering via caspase-3 activation [15]. Although, decorin is expressed in tumor stroma but it's rarely expressed by cancer cells and tissues themselves as has been demonstrated by analysis of a variety of tumors including colon, pancreas, prostate, lung, ovarian, breast cancer [16-19]. However, studies by us and others have shown that decorin is expressed by oral squamous cell carcinoma and osteosarcoma cells [20,21]. Osteosarcoma cells were shown to be insensitive to decorin-induced growth arrest, rather decorin seemed to be beneficial, since it was necessary for osteosarcoma cell migration [22].

Interleukin (IL)-8, a proinflammatory cytokine of the CXC chemokine family that was originally classified as a neutrophil chemoattractant, has been shown to play a significant role in tumor progression and metastasis in a variety of human cancers, including oral cancers [23,24]. This chemokine activates multiple intracellular signalling pathways downstream of two cell-surface, G protein coupled receptors; CXCR1 and CXCR2. IL-8 biologic activity in tumors and the tumor microenvironment contributes to cancer progression through its functions in the regulation of angiogenesis, cancer cell growth and survival, tumor cell invasion and metastasis, leukocyte infiltration and modification of immune responses [23,24]. Exogenous decorin has been shown to induce IL-8 production in endothelial cells [25].

Previously; we have shown that decorin is aberrantly expressed as well as translocated to the nucleus in dysplastic oral keratinocytes (DOK) and malignant squamous cell carcinoma (SCC-25) and in oral cancer biopsy tissue [20]. In the present study; using shRNA expression plasmid mediated *in vitro *stable gene silencing in DOK and SCC-25 cells, we demonstrate that nuclear localized decorin knockdown suppresses migration and invasion in dysplastic and malignant oral epithelial cells. Our data also indicates that reduced migration and invasion phenotype of these cells is mediated by IL-8 modulation as demonstrated by supplementation/rescue experiments. Furthermore, we show here that this nuclear localized decorin interacts with EGFR in the nuclear fractions of both DOK and SCC-25 cells, thus providing the first indication of a unique phenomenon in oral epithelial cells.

## Materials and methods

### Cell Lines and culture conditions

Oral epithelial origin, premalignant- Dysplastic Oral Keratinocyte (DOK) and malignant- Squamous Carcinoma Cell (SCC-25) lines were routinely maintained in DMEM/F12 (Hyclone, Logan, Utah) supplemented with 10% Foetal Calf Serum for use as *in vitro *model in our studies, as described previously [26,27]. DOK and SCC-25 cells (5 × 10^5 ^cells/well) were cultured in complete medium for 24 h, conditioned medium was harvested and was concentrated using a Centriplus centrifugal concentrator (Millipore, Bedford, Massachusetts).

### Decorin knock down in DOK and SCC-25 cells *in vitro*

Silencing of decorin gene expression was achieved using short hairpin RNA (shRNA) technology. Oligonucleotides targeting decorin transcript variants -A1 (RefSeq accession no NM_001920, at nucleotide position 720-740) and -A2 (RefSeq accession no NM_ 133503.2, at nucleotide position 566-586) and scrambled sequence non specific to any gene were custom synthesized, annealed, and cloned into the shRNA expression vector pGeneClip Puro™ (Promega) by Super Array Bioscience Corporation (Frederick, MD). BLAST queries were performed to ensure that the sequences have no significant homology with any other human genes. The transformation grade shRNAi plasmids were amplified in *E. coli *cultures, purified using Midiprep kits for endotoxin-free DNA vectors and then transfected into DOK and SCC-25 cells using Effectene™ transfection reagent following manufacturer's protocol (Qiagen, Valencia, CA). The stable transfectants were selected for puromycin (Calbiochem, San Diego, CA) antibiotic resistance at 2.5 μg/ml final optimal concentration. To avoid clone-specific variances, pools of stable transfectants (maintained at 1 μg/ml of puromycin) were used in all subsequent experiments. Decorin expression levels were determined at transcript and protein level by quantitative real-time reverse transcription-PCR (RT-PCR) and Western blotting, respectively. Hereafter, untransfected DOK and SCC-25 cells will be referred to as wild type (WT), scrambled shRNA stable transfectants as control (or Ctrl-shRNA in figures), and decorin shRNA stable transfectants as decorin silenced (or DCN-shRNA in figures).

### Real-time PCR

RNA was extracted from DOK and SCC-25 cells using RNeasy Plus mini kit (Qiagen, Valencia, CA) and 2.5 μg of total RNA was used to synthesize cDNA, using SuperScript III Reverse Transcriptase (Invitrogen, San Diego, CA). Quantitative RT-PCR was performed using QuantiTect™ SYBR Green PCR kit (Qiagen, Valencia, CA) on the Mini Opticon™ Real-Time PCR system (BioRad, Hercules, CA) as per manufacturer's protocol. Quantitative PCR primer pairs were designed for SYBR Green chemistry based detection of amplicons for *DCN *(5'-GGACCGTTTCAACAGAGAGG-3', 5'-GACCACTCGAAGATGGCATT-3'), *IL-8 *(5'-TCTGCAGCTCTGTGTGAAGG-3', 5'-TAATTTCTGTGTTGGCGCAG-3'), and *GAPDH *(5'-ACAGTCAGCCGCATCTTCTT-3', 5'-GTTAAAAGCAGCCCTGGTGA-3'). *GAPDH *was used as relative house- keeping gene expression control to normalize for sample variations.

### Cell proliferation assay

Cell proliferation was measured using CellTiter 96^® ^Aqueous One Solution -Cell Proliferation MTS based assay (Promega, Madison, WI) according to manufacturer's instructions. Briefly, WT, control and decorin silenced DOK and SCC-25 cells (10^5 ^cells/well), were cultured in 96-well flat-bottom plates at a final volume of a 100 μl for 24, 48, and 72 h. During the last hour of culture 20 μl of CellTiter 96^® ^Aqueous One Solution reagent, containing a tetrazolium compound [3- (4, 5-dimethylthiazol-2-yl) -5 - (3-carboxymethoxyphenyl)-2-(4-sulfophenyl)-2H-tetrazolium, inner salt; MTS] and an electron coupling reagent (phenazine ethosulfate; PES), was added to each well. Increase in absorbance at 490 nm wavelength (indicating cell proliferation) was measured using a 96-well plate reader (SPECTRAMax 190, Molecular Devices, Sunnyvale, CA) and results were analyzed by SOFTMax Pro software.

### Co-Immunoprecipitation and Western Blot Analysis

Cells were rinsed with ice-cold PBS and were lysed in a buffer containing 20 mM Tris, pH 7.6, 0.1% SDS, 1% Triton-X, 1% deoxycholate, 100 μg/ml PMSF, and protease inhibitor cocktail (Sigma-Aldrich, St. Louis, MO). Lysates were centrifuged at 20,000 × *g *for 20 min at 4°C. Nuclear extracts were prepared by using NE-PER kit reagents (Pierce, Rockford, IL) following manufacturer's protocol. Protein concentration was determined by Bis-Cinchonic Acid (BCA) protein assay (Pierce, Rockford, IL) and subjected to 10% SDS-PAGE analysis, followed by transfer to polyvinylidene difluoride membrane (Bio-Rad, Hercules, CA). The membranes were immunoprobed with 1:500 dilution of monoclonal anti-human decorin antibody (Abcam, Cambridge, MA) or 1:1000 dilution of anti-human β-tubulin polyclonal antibody. Western blots were developed with appropriate horseradish peroxidase conjugated secondary antibodies (Bio-Rad) and ECL Plus chemiluminescence system (Amersham, Arlington Heights, IL) and exposed to auto radiographic films. Radiographs were scanned and densitometry analysis was done using AlphaEase FC software (Alpha Innotech Corporation, San Leandro, CA). For analysis of nuclear lysates by coimmunoprecpitation, Dynabeads^® ^Protein G (Invitrogen, Carlsbad, CA) were used following manufacturer's protocol. Briefly, 5 μg of anti-decorin antibody was coupled to Dynabeads Protein G and then incubated with nuclear lysates to capture the target protein (decorin). Immunocomplexes were eluted and were resolved on 10% SDS-PAGE gel and analyzed by western blotting (as above) using decorin, EGFR antibodies (Abcam).

### ELISA for IL-8 Quantification

Decorin silenced, control and WT DOK and SCC-25 cells (5 × 10^5 ^cells/well) were cultured in complete medium in 24-well flat-bottom plates at a final volume of a 500 μl. Culture supernatants were collected and IL-8 was assayed with 100 μl of cell free culture supernatant using DuoSet IL-8 ELISA kit (R&D Systems, Minneapolis, MN) according to manufacturer's instructions. Absorbance was read at 450 nm with the SPECTRAMax 190 microplate spectrophotometer and results were analyzed by SOFTMax Pro software (Molecular Devices, Sunnyvale, CA). Sample concentrations were determined by interpolation from the standard curve. IL-8 detection limit was found to be 5.6 pg/ml. Samples were read in triplicate.

### Cell Migration and Invasion Assay

The ability of cells to migrate across control inserts (migration) or invade across Matrigel™- coated inserts (invasion) was assayed using BD Falcon control inserts or BD BioCoat Matrigel™ invasion chambers (BD Biosciences, San Jose, CA), respectively. The BD BioCoat Matrigel™ invasion chambers consist of BD Falcon tissue culture companion plate with Falcon cell culture inserts containing 8 micron pore size PET membrane, pre-coated with a thin layer of Matrigel™ basement membrane matrix. Manufacturer's instructions were followed to perform the assay. Briefly, serum free DMEM/F12 medium (0.5 ml) containing 10^5 ^cells were added to the upper chamber, and 0.75 ml of DMEM/F12 medium containing 10% serum was added to the lower chamber as a chemo-attractant. After overnight incubation at 37°C and 5% CO2, cells on the upper surface of the filter (cells that had not penetrated the filter) were removed using a cotton swab. Cells that had migrated to the lower surface of the filter were fixed in 100% methanol and stained with 0.005% crystal violet. For each filter, the number of migrated cells in 5 medium-power fields (magnification of 20×) was counted using bright field microscopy, and photographed. Assays were performed in duplicates and repeated at least three times. Invasion index is expressed as percentage of invading cells, and is calculated by dividing mean number of cells invading through Matrigel™ membrane over mean number of cells migrating through the non-coated control insert membrane per microscopic filed over five fields per assay, and ratio then multiplied by 100 for percent values. 

### Statistical Analysis

Student's paired *t *test was used to determine the statistical significance of the data. Statistical analysis was performed on Graph Pad Prism Software. Significance was evaluated at *p *values: * *p *< 0.05, ** *p *< 0.01, *** *p *< 0.001.

## Results

### Stable knockdown of decorin using shRNA in DOK and SCC-25

**Figure 1:**
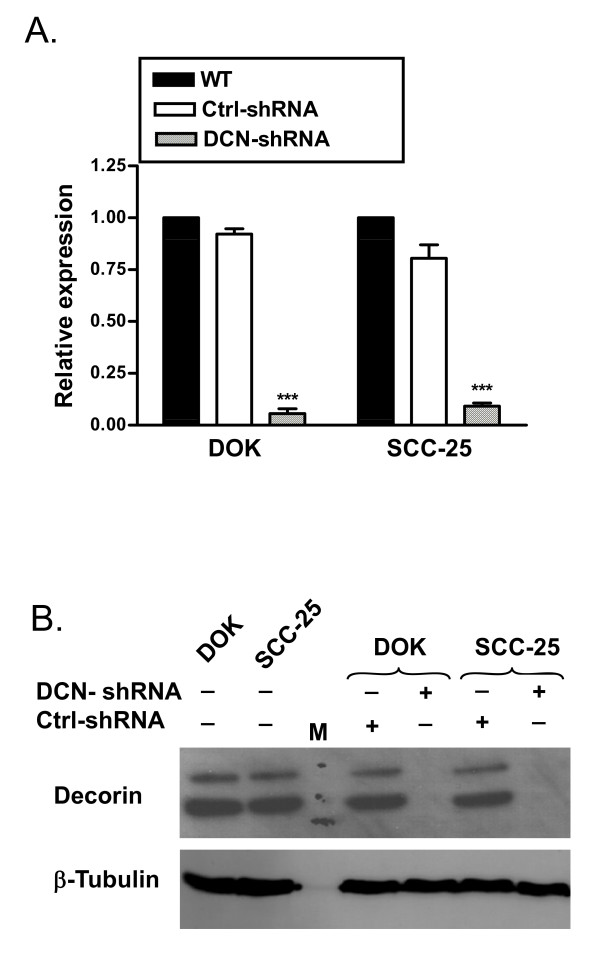
**Validation of stable knockdown of decorin in DOK and SCC-25 cells**. DOK and SCC-25 cells were stably transfected with decorin-shRNA (DCN-shRNA), or scrambled sequence-shRNA (Ctrl-shRNA) or no transfection control (WT). *A*, RNA was extracted and cDNA was subjected to quantitative RT-PCR, normalized decorin expression from one representative experiment of three. *B*, Nuclear lysates were extracted and were subjected to SDS-PAGE followed by immunoblotting with anti-decorin and anti-β-tubulin antibodies. Data presented is one representative immuoblot of at least three experiments. *** *p *< 0.001 compared to respective controls.

To study the functional role of aberrantly expressed nuclear decorin in dysplastic and malignant epithelial cells, decorin shRNA-stable clones were generated. Briefly, DNA oligonucleotides specific for decorin mRNA target sequence and a non-gene scrambled control were ligated into pGeneClip™ Puro plasmid, and are herein referred to as decorin shRNA (DCN-shRNA) and control shRNA (Ctrl-shRNA), respectively. DOK and SCC-25 cells were transfected with these constructs and puromycin resistant positive stable clones were selected. To avoid clone-specific effects, pooled transfectants were used for each cell type. Knockdown of decorin expression was confirmed by real-time PCR and western blot analysis. Pooled decorin-shRNA transfected DOK clones showed a significant (more than 80%) decrease in decorin mRNA expression when compared to control-shRNA transfected clones or no transfection wild type DOK (Figure 1A). Similar results were observed in SCC-25 cells (Figure 1A). Decorin knock down was also confirmed by western blot. Pooled decorin-shRNA transfected DOK or SCC-25 clones showed almost complete abrogation of decorin protein expression in nuclear lysates (Figure 1B). Similar decorin protein expression knock down was observed in whole cell lysates (data not shown). These results demonstrate that decorin-shRNA successfully silenced the nuclear decorin expression in DOK and SCC-25 cells.

### Decorin knockdown does not affect cell proliferation in dysplastic and malignant epithelia

**Figure 2:**
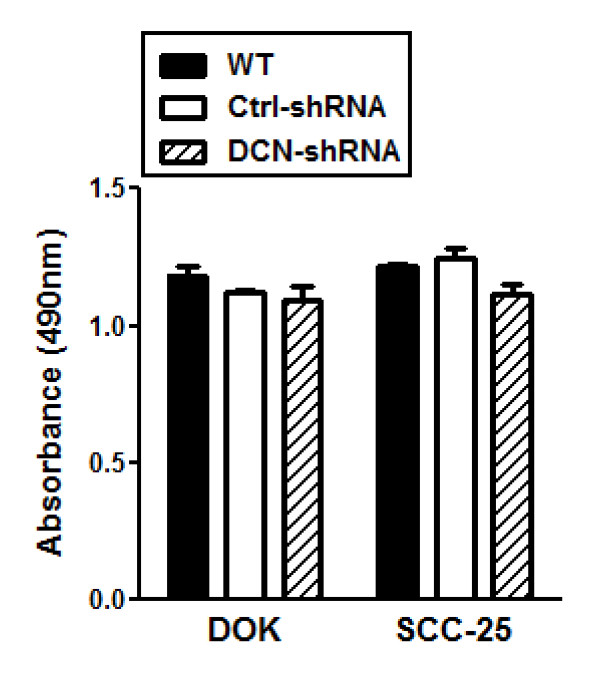
**Decorin silencing does not affect DOK or SCC-25 cell growth/proliferation**. WT, control and decorin silenced DOK and SCC-25 cells were cultured for 24 h. During the last hour of culture, 20 μl of CellTiter 96^® ^Aqueous One Solution Reagent containing a tetrazolium compound [3-(4,5-dimethylthiazol-2-yl)-5-(3-carboxymethoxyphenyl)-2-(4-sulfophenyl)-2H-tetrazolium, inner salt; MTS] and an electron coupling reagent (phenazine ethosulfate; PES) was added to the media (100 μl per well), and color changes were recorded by absorbance at 490 nm. Data are presented as mean ± SE of three replicates of one representative experiment of three.

To evaluate the effect of aberrantly expressed nuclear decorin on the cellular proliferation rates of dysplastic and malignant oral epithelial cells, DOK and SCC-25 WT cells, DCN-shRNA transfectants and ctrl-shRNA transfectants were allowed to grow in culture for 24, 48 and 72 h and proliferation was assessed by MTS assay. Compared with WT or control-shRNA cells, decorin silenced DOK and SCC-25 cells did not show any change in cell proliferation rates at 24 hrs (Figure 2). Similar results were obtained at 48 and 72 h time points (data not shown). This might be due to sequestration of decorin in the nucleus and consequent inability to interact with membrane receptors to which extracellular decorin is known to bind to effect tumor growth in other cancers.

### Decorin knockdown mitigates migratory and invasive phenotype of dysplastic and malignant oral epithelial cells

**Figure 3:**
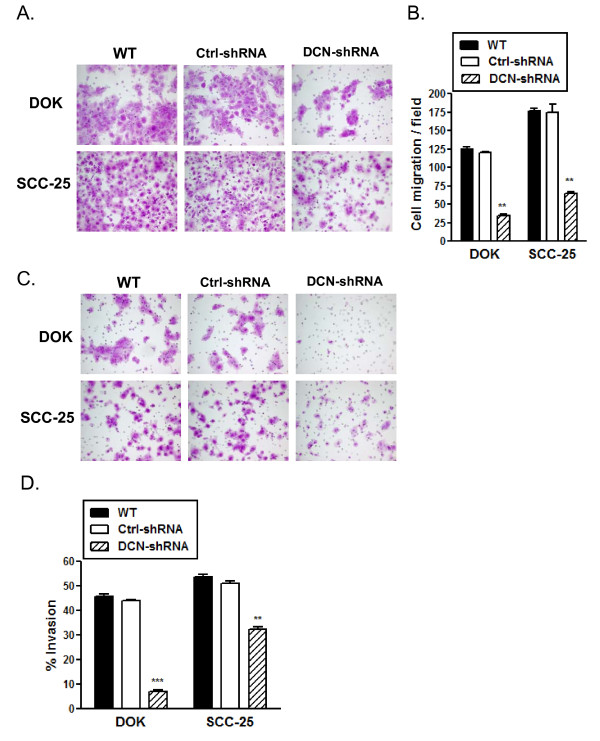
**Migration and invasion suppression in decorin silenced cell lines**. *A*, Cell motility through uncoated filters (migration) was measured 22 h after plating. The migrating cells were fixed, stained, and photographed as described in materials and methods. Each panel represents one representative field of five from duplicate filters of three experiments. *B*, Migrated cells in each one of the five fields of duplicate filters were counted, numbers represent mean ± SD of three experiments. *C*, Cells that invaded across the Matrigel™ layer were fixed, stained, and photographed. Each panel represents one representative field of five from duplicate filters of three experiments. *D*, Migrated and invaded cells in five fields of duplicate filters were counted and % invasion was calculated as described in materials and methods. Numbers represent mean ± SD of three individual experiments. ** *p *< 0.01, *** *p *< 0.001 compared to respective controls.

We examined whether decorin silencing has any effect on migration and invasion properties of dysplastic and malignant oral epithelial cells. Using an *in vitro *trans well assay and 10% FBS as a chemo-attractant, we observed a significant suppression of cell migration in both decorin-silenced DOK and SCC-25 cells compared to respective WT or control cells (Figure 3A &3B). Next, we determined the invasive property of these cells as measured through invasion across a Matrigel™ impregnated porous (8 μm) membrane. Invasive phenotype was observed to be significantly suppressed in decorin-silenced SCC-25 cells and was almost completely abrogated in decorin-silenced DOK cells (Figure 3C &3D). Similar results were obtained when conditioned media from DOK WT was used as a chemo-attractant (data not shown). It is important to note that overall malignant SCC-25 cells have relatively higher migration and invasion rates than the premalignant and dysplastic DOK cells.

### Attenuation of IL-8 production in decorin silenced DOK and SCC25 cells

**Figure 4:**
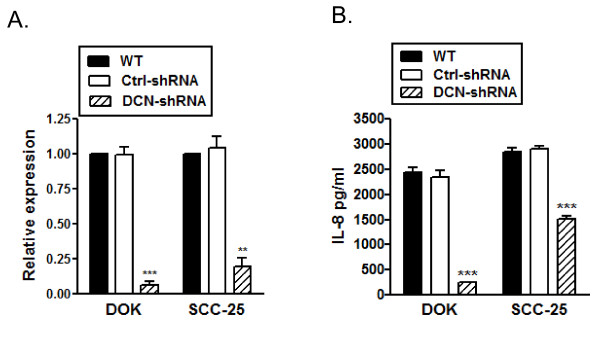
**Reduced IL-8 production in decorin Silenced DOK and SCC25**. *A*, RNA was extracted from WT, control and decorin silenced DOK and SCC-25 cells and cDNA was subjected to quantitative RT-PCR, normalized IL-8 expression from one representative experiment of three. *B*, Cells were cultured and IL-8 was measured in 24 h culture supernatants using ELISA. Data are presented as mean ± SD of three replicates of one representative experiment of four. ** *p *< 0.01, *** *p *< 0.001 compared to respective controls.

IL-8 is an important pro-inflammatory chemokine and is involved in tumor progression in a variety of malignancies. In particular, IL-8 has been shown to induce migration and invasion in oral squamous cell carcinoma cell lines[23]. Moreover, decorin has been shown to induce IL-8 production in endothelial cells [25]. Therefore, we sought to determine if nuclear decorin silencing has an effect on IL-8 production in these dysplastic and malignant oral epithelial cells. Interestingly, IL-8 expression was significantly reduced in nuclear decorin-silenced DOK or SCC-25 cells as compared to the control and WT cells. Real-time PCR analysis revealed over 90% reduction in constitutive IL-8 expression in decorin-silenced DOK and about 70% reduction in decorin-silenced SCC-25 cells (Figure 4A). IL-8 protein production, as measured by ELISA, was found to be significantly reduced in decorin-silenced DOK and SCC-25 cells (Figure 4B). However, as observed with IL-8 expression levels, the effect of decorin silencing on IL-8 production was more pronounced in DOK than in SCC-25 cells.

### Supplementation with IL8 rescues migration and invasion suppression in decorin silenced cell lines

**Figure 5:**
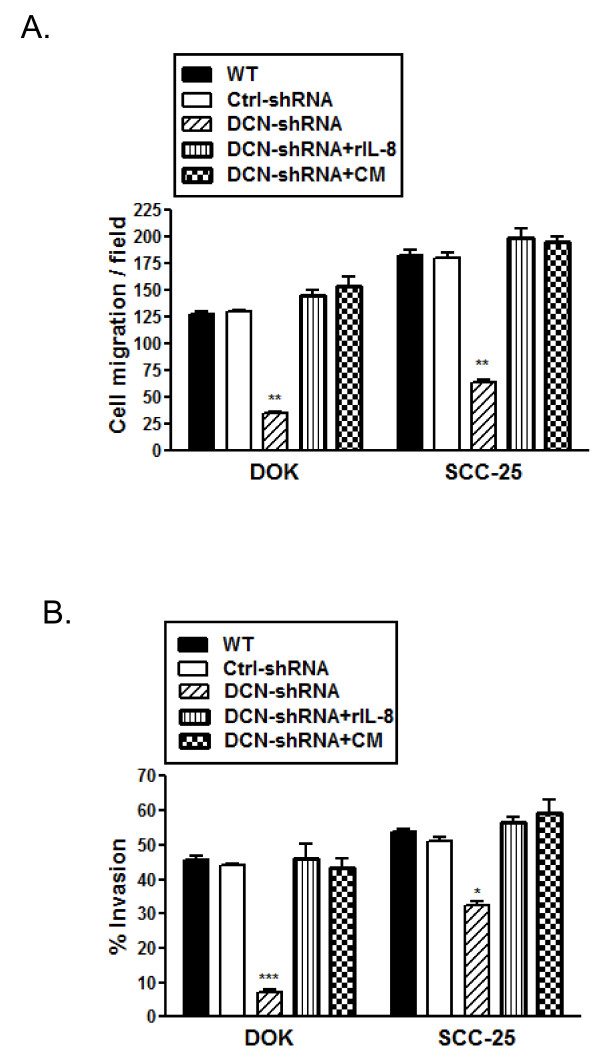
**Migration and invasion suppression rescued by addition of IL8 in decorin silenced cell lines**. Decorin silenced DOK and SCC-25 cells were cultured with 2.5 ng/ml of recombinant human IL-8 (R&D Systems, Minneapolis, MN) or conditioned medium (CM) from respective wild type cell lines for 24 h prior to migration and invasion assay. *A*, Cell motility through uncoated filters (migration) was measured 22 h after plating. The migrating cells were fixed, stained, and photographed as described in materials and methods. *A*, Migrated cells in each one of the five fields of duplicate filters were counted, numbers represent mean ± SD of three experiments. *B*, Cells that invaded across the Matrigel™ layer were fixed, stained, and photographed. Migrated and invaded cells in five fields of duplicate filters were counted and % invasion was calculated as described in materials and methods. Numbers represent mean ± SD of three individual experiments. * *p *< 0.05, ** *p *< 0.01, *** *p *< 0.001 compared to respective controls.

Next, we sought to determine whether diminished migration and invasion in decorin-silenced DOK and SCC-25 cells was due to reduced IL-8 expression. Decorin silenced DOK and SCC-25 cells were cultured with 2.5-5.0 ng/ml of recombinant human IL-8 (rIL-8) or conditioned medium from respective un-transfected (wild type) cell lines for 24 h prior to migration and invasion assay. As shown in Figure 5A and 5B, both migratory and invasive phenotype of decorin silenced dysplastic and malignant oral epithelial cells was salvaged by pre-incubation with rIL-8 or IL-8 containing conditioned medium emphasising the role played by this cytokine in this phenomenon. Similar results were obtained for 5.0 ng/ml rIL-8 (data not shown).

### Decorin is associated with EGFR in the nuclear compartment

**Figure 6:**
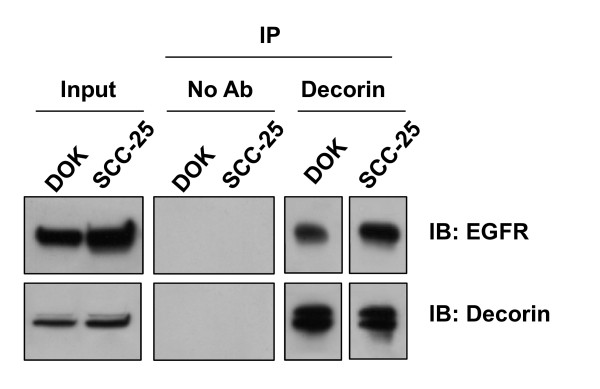
**Nuclear Decorin associates with EGFR**. Nuclear lysates were extracted from DOK and SCC-25 cells and were immunoprecipitated (IP) with decorin antibody. Immunocomplexes were subjected to SDS-PAGE and were analysed by immunoblotting (IB) with anti-decorin and anti-EGFR antibodies.

We have previously shown that decorin is aberrantly expressed and localized to the nucleus in DOK and SCC-25. However, decorin is not known to carry nuclear localization signal. In an effort to identify possible binding partners of decorin in the nucleus of these dysplastic and malignant oral epithelial cells, we explored weather nuclear decorin physically associates with nuclear EGFR. Nuclear lysates were obtained from DOK and SCC-25 cells. Interestingly, immunoprecipitation with decorin antibody co-precipitated EGFR in both dysplastic (DOK) and malignant (SCC-25) oral epithelial cells (Figure 6). However, similar immunoprecpitation with decorin antibody did not co-precipitate either biglycan or LRRFIP2, the other two suspect NLS carrying proteins (data not shown). Our results suggest that nuclear localized decorin interacts with nuclear localized EGFR in these dysplastic and malignant oral epithelial cells. Intriguingly, EGFR (trans) activation has been shown to mediate IL-8 production in epithelial cells [28,29].

## Discussion

Decorin, a multifunctional molecule of the extracellular matrix, has been assigned a plethora of functions. Among these, the most intriguing is the ability to inhibit growth and metastasis of a wide range of cancer cells in vitro. Functions of decorin as a naturally occurring oncosuppressive agent are being studied in detail. Decorin is normally present in the extracellular stromal compartment and analysis of a variety of tumours indicates that it is seldom expressed by cancer tissue. However studies by us and others have demonstrated that there are exceptions to this prevailing decorin expression and decorin mediated growth suppression model in cancer. In our previous studies of oral precancerous and cancerous lesions and cellular models of oral cancer progression, we demonstrated that decorin is aberrantly expressed and localized in the nucleus in the dysplastic and malignant oral epithelial cells [20]. In this study we used shRNA expression plasmid mediated *in vitro *stable gene silencing in DOK and SCC-25 cells to study the role of nuclear localized decorin in oral cancer.

The ability of a cancer cell to undergo migration and invasion allows it to change position within the tissues allowing neoplastic cells to enter lymphatic and blood vessels for dissemination into the circulation, and then undergo metastatic growth in distant organs. Using an *in vitro *Matrigel™ coated and uncoated Trans well chamber based invasion and migration assay, we find here that nuclear decorin silencing results in impaired invasive and migratory potential in both dysplastic and malignant oral epithelia. It is interesting to note that in osteosarcoma cells, decorin was also found to be necessary for cell migration [21]. Moreover, these Osteosarcoma cells have also been shown to constitutively produce decorin and manifest resistance to decorin induced growth arrest.

Growth and motility of various cancer cells is influenced by assorted growth factors, cytokines and chemokines. Recently, there has been increasing evidence that chemokines have a role in tumor biology. Chemokines were first described as small peptides controlling cell migration, especially that of leukocytes during inflammation and immune response. Since then, a broad spectrum of biological activities has been described as chemokine-regulated tumorigenesis that effect tumors and their microenvironment. Increased expression of IL-8 and/or its receptors has been characterized in cancer cells, endothelial cells, infiltrating neutrophils, and tumor-associated macrophages, suggesting that IL-8 may function as a significant regulatory factor within the tumor microenvironment. Many tumor cells, such as prostate carcinoma, melanoma, breast carcinoma and gastric carcinoma cell lines, respond chemotactically to IL-8 [30-34]. IL-8 can also act as an autocrine growth factor for melanoma, colon carcinoma, lung adenocarcinoma and prostate carcinoma cell lines [35-38]. In addition, IL-8 has been shown to be produced by oral squamous cell carcinoma both constitutively and in response to cytokines[23]. In these oral carcinoma cell lines, IL-8 was shown to have positive influence on migration and invasion via up-regulation of MMP-7. Given the importance of IL-8 in tumor migration and invasion, we tested whether IL-8 was involved in nuclear decorin silencing mediated migration and invasion suppression in our system. We found that, IL-8 production was significantly attenuated in nuclear decorin silenced DOK and SCC25 cells. Rescue of migration and invasion suppression upon addition of exogenous IL8 indicates that reduced IL-8 might be forming the basis of migration and invasion mitigation in these decorin silenced cell lines. Whether or not there is a direct or indirect relationship between decorin and IL-8 is not known currently and needs further exploration. However, intriguingly, decorin has been shown to induce IL-8 production at least in endothelial cells [25].

We have previously shown that decorin is aberrantly expressed in the nucleus in DOK and SCC-25 oral cell lines and human biopsy tissues [20]. However, underlying mechanism by which decorin localizes to the nucleus in these cells is unclear. Decorin lacks nuclear localization signal (NLS), hence it might be binding with another protein that carries NLS thus providing a means for decorin to localize to the nucleus in the oral cells. Elucidation of potential interactions of the aberrant decorin expressed in the oral cancer progression model and its biological implications need to be addressed further. As such, in this study we explored the possible nuclear decorin binding partners by immuoprecipitation experiments on nuclear lysates obtained from DOK and SCC-25 cells. Decorin is a known alternate biological ligand for EGFR, other than EGF [11]. It binds to a discrete region of the EGFR which is partially overlapping but distinct from the EGF-binding domain [12]. Emerging evidence suggests that EGFR family carries NLS and either full length or fragmented EGFR family members can be shuttled from the plasma membranes to the nucleus [39-41]. This direct mode of the EGFR signaling pathway is distinct from the traditional transduction pathway and involves cellular transport of EGFR from the cell-surface to the cell nucleus, association of nuclear EGFR with transcriptional co-factors and target gene promoters, and transcriptional regulation of the target genes. Aberrant expression and nuclear localization of EGFR has been demonstrated by many different groups in a variety of human cancers including oral cancer [42-44]. Mutant EGFR, a truncated EGFR variant (EGFRvIII), has also been described in head and neck cancer [45]. In search of a possible mechanism by which decorin is aberrantly localized in the nucleus, we sought out to determine if nuclear decorin interacts with nuclear EGFR in our oral cancer model. We found that EGFR is expressed in the nuclear lysates obtained from DOK and SCC-25 cells, and very interestingly, results of our co- immunoprecipitation experiments demonstrate that nuclear decorin does interacts with nuclear EGFR in these dysplastic and malignant oral epithelia. This indicates a possible means by which decorin is aberrantly localized in the nuclei in our oral cancer model. As mentioned above, nuclear EGFR has been shown to act as transcription factor and its transcriptional activity appears to depend on its C-terminal transactivation domain and its physical and functional interaction with other transcription factors that contain DNA-binding activity [46]. Interestingly, EGFR (trans) activation has been shown to be involved in IL-8 production in lung and skin epithelia [28,47]. Whether or not nuclear EGFR has direct or indirect role in IL-8 transcription is not known currently. Besides decorin-EGFR relation, additional interactions with other nuclear and/or cytosolic factors may result from the aberrant localization of decorin and perhaps result in some of decorin's tumor-promoting role in oral cancer. Together, results from our study suggest the importance of decorin as a potential therapeutic target in oral cancer, as it modulates migration and invasion of premalignant and malignant oral epithelial cells. Further mechanistic studies are warranted to decipher how IL-8 expression is regulated by nuclear localization of decorin and/or its interaction with EGFR in these cells. Studies in our laboratory are underway in this direction which will shed light on additional biological aspects of nuclear localized decorin in oral cancer progression.

## Conclusions

Using shRNA expression plasmid mediated *in vitro *stable gene silencing in dysplastic and malignant oral epithelial cells; we present here a study of the role of nuclear localized decorin in oral cancer progression. We demonstrate that nuclear localized decorin knockdown suppresses migration and invasion in DOK and SCC-25 cells. Our data also indicate that reduced migration and invasion phenotype of these cells is perhaps mediated by IL-8 modulation. Furthermore, we show here that nuclear localized decorin interacts with nuclear EGFR in this oral cancer cell line model. The results of our study highlight the importance of decorin as a potential oral cancer specific therapeutic target.

## Competing interests

The authors declare that they have no competing interests.

## Authors' contributions

ND contributed to study concept and design and devised, performed, and analyzed the experiments, interpreted data and wrote the manuscript. AGB conceptualized the study design, analyzed results and helped in data interpretation, approved the final manuscript and directed the research program. All authors reviewed and approved the final manuscript.
